# Cardioprotective Properties of Omecamtiv Mecarbil against Ischemia and Reperfusion Injury

**DOI:** 10.3390/jcm8030375

**Published:** 2019-03-18

**Authors:** Martin Stroethoff, Friederike Behmenburg, Simon Meierkord, Sebastian Bunte, Felix Mayer, Alexander Mathes, André Heinen, Markus W. Hollmann, Ragnar Huhn

**Affiliations:** 1 Department of Anesthesiology, University Hospital Duesseldorf, Moorenstr. 5, 40225 Duesseldorf, Germany; Martin.Stroethoff@med.uni-duesseldorf.de (M.S.); Friederike@Behmenburg.de (F.B.); Simon.Meierkord@uni-duesseldorf.de (S.M.); Sebastian.Bunte@med.uni-duesseldorf.de (S.B.); 2 Department of Forensic Medicine, University Hospital Duesseldorf, Moorenstr. 5, 40225 Duesseldorf, Germany; Felix.Mayer@med.uni-duesseldorf.de; 3 Department of Anesthesiology and Intensive Care Medicine, University Hospital Cologne, Kerpener Str. 62, 50937 Cologne, Germany; Alexander.Mathes@uk-koeln.de; 4 Institute of Cardiovascular Physiology, Heinrich-Heine-University Duesseldorf, Universitaetsstr. 1, 40225 Duesseldorf, Germany; Andre.Heinen@med.uni-duesseldorf.de; 5 Department of Anesthesiology, Amsterdam University Medical Center (AUMC), Location AMC, University of Amsterdam, Meiberdreef 9, 1100DD Amsterdam, The Netherlands; M.W.Hollmann@amc.uva.nl

**Keywords:** omecamtiv mecarbil, myocardial infarction, preconditioning, postconditioning

## Abstract

Omecamtiv mecarbil (OM) is a first-in-class myosin activator. It was developed as a new inotropic therapy option for heart failure and is currently the object of a phase 3 clinical trial program. OM activates ryanodine receptors, which were shown to be involved in cardioprotection induced by conditioning strategies. We hypothesize that OM exerts a concentration-dependent cardioprotective effect through pre- and postconditioning. Isolated male Wistar rat hearts underwent 33 min of global ischemia and 60 min of reperfusion. OM was administered in various concentrations (1, 3, 10, and 30 µM) over 10 min prior to ischemia. Based on these results, in subsequent experiments 3 and 10 µM OM were given over 10 min after ischemia. Infarct sizes were determined by TTC staining. In controls, the infarct size was 60% ± 10% and 59% ± 12%, respectively. Ten micromolar OM before ischemia reduced the infarct size to 33% ± 8%. The lower concentrations did not initiate cardioprotection, and the next highest concentration did not enhance the protective effect. Even if 10 μM OM was given in the early reperfusion phase, it significantly reduced the infarct size (31% ± 6%), whereas 3 μM OM did not trigger a protective effect (58% ± 15%). This study shows for the first time that OM induces cardioprotection by pre- and postconditioning with a binary phenomenon, which is either ineffective or has a maximal effect.

## 1. Introduction

According to current figures of the Center for Disease Control and Prevention (CDC), every 40 s someone in the United States has a myocardial infarction, making this disease one of the leading causes of death [1]. The most important strategy in the case of acute myocardial ischemia is the immediate reperfusion to preserve the cardiac tissue. Paradoxically, reperfusion itself also leads to additional myocardial damage. Ischemia-reperfusion injury (I/R injury) can be manipulated to reduce the impact. Both the phenomenon of ischemic preconditioning, first described by Murry et al. [2], and ischemic postconditioning, discovered by Zhao et al. [3], demonstrated that short sub-lethal ischemic periods before or after prolonged ischemia provide increased myocardial tolerance to I/R injury and reduced infarct size. In addition to these ischemic stimuli, it is also possible to mimic these cardioprotective interventions by pharmacological agents like sildenafil [4] or milrinone [5].

Omecamtiv mecarbil (OM, CK-1827452) is a selective small-molecule activator of cardiac myosin and was developed as a new inotropic therapy option for heart failure [6,7]. It is currently the subject of a phase 3 clinical trial program [8]. OM increases the total number of myosin heads bound to actin filaments, prolongs the systole duration, and thus provides a stronger cardiac contraction without affecting cardiomyocyte intracellular calcium accumulation [9,10,11]. It has been shown that OM activates cardiac ryanodine receptors [12]. The activation of these receptors is involved in cardioprotection induced by pre- and postconditioning [13,14]. So far, it is not known if OM has cardioprotective potency and is able to reduce I/R injury.

The aim of the present study was to examine whether (1) OM induces cardioprotection by pre- or postconditioning and (2) if the effect is concentration-dependent. 

## 2. Material and Methods

The study was approved by the Animal Ethics Committee of the University of Duesseldorf, Germany, and conducted in accordance with the Guide for the Care and Use of Laboratory Animals of the National Institutes of Health [15].

The surgical preparation of male Wistar rats was performed as described previously [5]. In brief, rats were anesthetized by intraperitoneal injection of 90 mg/kg pentobarbital. Hearts were excised after thoracotomy, mounted on a Langendorff system, and perfused at a constant pressure of 80 mmHg with a Krebs-Henseleit solution (KHS). The solution was oxygenated with carbogen (95% O_2_ and 5% CO_2_) and composed of (in mM) 118 NaCl, 4.7 KCl, 1.2 MgSO_4_, 1.17 KH_2_PO_4_, 24.9 NaHCO_3_, 2.52 CaCl_2_, 0.5 EDTA, 11 glucose, and 1 lactate at 37 °C. Into the left ventricle, a fluid-filled balloon was inserted to set an end-diastolic pressure at 3–8 mmHg. During the experiments, we measured heart rate, left ventricular end-diastolic pressure (LVEDP), and coronary flow. These values were digitized at a sampling rate of 500 Hz through the use of an analog to digital converter system (PowerLab/8SP; ADInstruments Pty Ltd, Castle Hill, Australia). Data were continuously recorded on a personal computer using Chart for Windows v5.0 (ADInstruments Pty Ltd, Castle Hill, Australia). The maximal contracture and the time to maximal contracture were detected during index ischemia.

### 2.1. Experimental Protocol

The study was designed in two cohorts. Each group underwent a baseline period of 20 min, 33 min of global ischemia, and 60 min of reperfusion, respectively. Global ischemia was achieved by stopping coronary perfusion of the heart with KHS.

**Figure 1:**
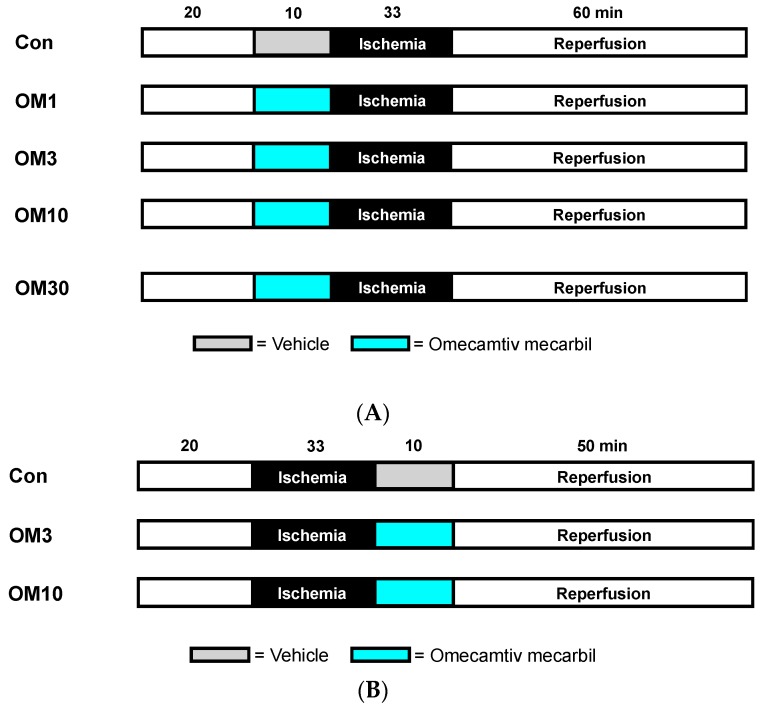
Experimental protocols. (**A**) Investigation of OM preconditioning. Con = control, OM = omecamtiv mecarbil (1–10 µM). (**B**) Investigation of OM postconditioning. Con = control, OM = omecamtiv mecarbil (3–10 µM).

The first cohort was designed to investigate the preconditioning effect of OM and to find the lowest cardioprotective concentration of OM. Different concentrations of OM (1, 3, 10, and 30 µM) were administered over a time period of 10 min before global ischemia (Figure 1A). Initially, rat hearts were randomly assigned into four groups (*n* = 8 per group): 

**Control (Con):** Hearts were perfused with Krebs-Henseleit solution for 10 min.

**Omecamtiv mecarbil (OM):** Hearts were perfused with 1, 3, and 10 µM OM for 10 min.

To test whether the effect of 10 µM OM can be enhanced, we investigated the concentration of 30 µM OM (*n* = 6). 

The second cohort was performed to determine whether OM could induce cardioprotection by postconditioning. We used the lowest cardioprotective concentration of OM (10 µM), which was detected in the first cohort, and the next lower concentration (3 µM). OM was given over a time period of 10 min at the onset of reperfusion after global ischemia (Figure 1B). Rat hearts were randomly assigned to three groups (*n* = 8 per group): 

**Control (Con):** Hearts were perfused with Krebs-Henseleit solution for 10 min.

**Omecamtiv mecarbil (OM):** Hearts were perfused with 3 and 10 µM OM for 10 min.

After reperfusion, hearts were cut into transverse slices, which were stained with 0.75% triphenyltetrazoliumchloride (TTC) solution. The size of the infarcted area was determined by planimetry using the SigmaScan Pro 5^®^ computer software (SPSS Science Software, Chicago, IL, USA).

### 2.2. Statistical Analysis

All data are expressed as mean ± SD. Statistical data were analyzed by using GraphPad StatMate^TM^ (GraphPad Software, San Diego, CA, USA). The calculated sample size was *n* = 8 for detecting a 25% mean difference in infarct size (power 80%, α < 0.05 (two–tailed)). A researcher blinded to the experimental groups evaluated the infarct sizes. Infarct sizes were analyzed by one–way ANOVA followed by Tukey’s post-hoc test. Comparisons of hemodynamic data among groups or among time points in a group were analyzed by two–way ANOVA followed by Tukey’s post-hoc test for group effects. Changes were considered statistically significant if *P* values were less than 0.05. 

## 3. Results

### 3.1. Animal Characteristics

In both parts of the study, there were no differences in body weight or heart weight between the different experimental groups (Table 1 and Table 2). In the preconditioning part, OM concentrations of 3, 10, and 30 µM demonstrated statistically different values of time and level of maximal ischemic contracture compared to the Con and OM1 group (Table 1). In the postconditioning part, there were no differences in time and level of maximal contracture between the experimental groups (Table 2).

### 3.2. Infarct Size

**Figure 2:**
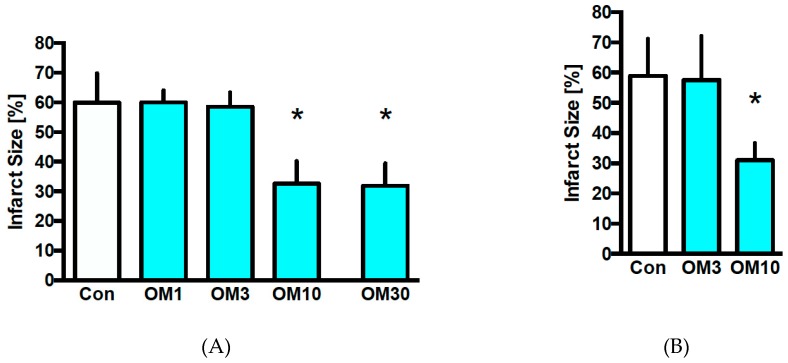
Infarct size measurement. (**A**) Infarct size of controls (Con) and preconditioning with omecamtiv mecarbil (OM). (**B**) Infarct size of controls (Con) and postconditioning with omecamtiv mecarbil (OM) * *p* < 0.05 vs. Con.

In the first cohort investigating the preconditioning effect, the infarct size of the control group was 60% ± 10% (Figure 2A). OM at 10 µM was the lowest cardioprotective concentration with an infarct size of 33% ± 8% (*p* < 0.05 vs. Con). A higher concentration of 30 µM OM induced comparable infarct size reduction as 10 µM (OM30: 32% ± 8%; *p* < 0.05 vs. Con and ns vs. OM10). Lower concentrations of OM at 1 and 3 µM did not reduce infarct size (OM1: 60% ± 4%, OM3: 59% ± 5%; each ns vs. Con). 

Based on the results of the first cohort, we used OM at 3 and 10 µM for investigating postconditioning effects. In the control group, the infarct size was 59% ± 12% (Figure 2B). OM at 10 µM reduced the infarct size to 31% ± 6% (*p* < 0.05 vs. Con). Like in the first cohort, the lower concentration with 3 µM OM did not reduce infarct size (OM3: 58% ± 15%; ns vs. Con).

### 3.3. Hemodynamics

In both parts of the study, reperfusion increased left ventricular end-diastolic pressure (LVEDP) and decreased coronary flow (all *p* < 0.05 vs. baseline) within the respective groups, while heart rate was mostly unchanged (Table 3 and Table 4). During baseline OM concentrations of 10 and 30 µM and preconditioning OM concentrations of 1 and 3 µM, an increased coronary flow compared to the control group (*p* < 0.05) in the preconditioning part was seen. Furthermore, OM concentrations of 3, 10, and 30 µM increased LVEDP before the global ischemia period compared to Con and 1 µM OM (*p* < 0.05; Table 3). Also, in these groups, the time to maximal ischemic contracture was delayed and the level of maximal ischemic contracture was lower (*p* < 0.05 vs. Con and OM1; Table 1). This effect was not detected in the postconditioning part, as OM was administered at the beginning of the reperfusion period and the treatment until reperfusion was the same in all groups (Table 2). There were no significant differences in the body and heart weight in both parts of the study (Table 1 and Table 2).

## 4. Discussion

The results of this study show for the first time that Omecamtiv mecarbil (OM), a first-in-class cardiac myosin activator, induces cardioprotection by pre- and postconditioning in the isolated perfused rat heart. The lowest cardioprotective concentration of OM was 10 µM for both cardioprotective interventions.

OM is a cardiac myosin activator and was developed as a new therapy option for heart failure. Heart failure is still a leading public health problem with an increasing myocardial contractility impairment and compromised cardiac output. However, the inotropic agents available so far failed to fulfill therapeutic expectations and hence the need for new agents like OM. Myocardial infarction is still the most common cause of heart failure [16], and in the future OM will likely be used in this endangered patient cohort. Accordingly, we hypothesized that OM might have infarct size-reducing effects besides its benefit in heart failure.

The idea of ischemic preconditioning is conceivably difficult for clinical routines. Postconditioning intervention would be more practical. Nevertheless, our initial examination in the first cohort was designed to study the cardioprotective capacity of OM by preconditioning. We wanted to find the lowest cardioprotective concentration to exclude possible overstimulation with potential side effects by an overly high concentration of OM. Our results demonstrate that below 10 µM there is no infarct size reduction, and with the administration of 30 μM we were able to exclude a possible concentration-dependent effect. OM at 30 µM did not provide a stronger cardioprotective effect demonstrating an on–off phenomenon of OM induced cardioprotection.

As mentioned above, postconditioning is the more interesting intervention for clinical practice as myocardial ischemia is mostly not predictable. We could show that 10 µM OM is cardioprotective even when applied in the early reperfusion phase, whereas 3 µM did not trigger an infarct size reduction. As OM was administered at the beginning of the reperfusion period after global ischemia, we could not detect hemodynamic differences during the 10 min of application between the groups. Thus, OM might be an option for treatment strategies for the failing heart after myocardial infarction.

The binding site of OM is the amino acid serine 148 in cardiac myosin. This reversible binding causes allosteric changes and modulates the myosin activity so that the speed of ATP hydrolysis and P_i_ release, a rate-limiting step, is enhanced. This step is a central process in cardiac force development, and the enhancement leads to an increased number of myosin heads bound to actin filaments and improved force production without cytosolic calcium accumulation. Thus, OM increases the number of force-generating cross bridges and prolonging the duration of the systole. In previous clinical studies, the prolongation of systole at the expense of diastole and the development of myocardial ischemia seemed to be a concentration-limiting factor. According to the ATOMIC–AHF study, a peak plasma concentration of 0.25–1.25 µM was well tolerated by the patients, whereas in a dose-ranging phase 2 trial, concentrations of 3–4 µM triggered symptoms of myocardial infarction [17]. These symptoms were attributed to the excessive prolongation of the systole, and this could also be a possible explanation of the preconditioning effect in our model. The shortened diastole could be protective by mimicking a kind of ischemic preconditioning. Interestingly, an infarct size reduction was only observed in concentrations of OM that dramatically increased LVEDP suggesting a correlation between the infarct size-reducing effects of OM on the one hand and the hemodynamic side effects, i.e., increase in LVEDP, on the other hand. It is important to note that the administration of OM in concentrations that dramatically increased LVEDP, and presumably shortened the duration of the diastole, did not affect coronary flow to a larger extent as observed. The reason for this is unclear, and we can only speculate that metabolic control of the coronary vasculature caused vasodilation resulting in a normalized coronary flow. Therefore, regional flow distribution and myocardial oxygen consumption under OM administration have to be investigated in more detail.

Although in previous studies no increased oxygen consumption was described under OM, a recent study from Bakkehaug et al. showed that there is significantly increased oxygen consumption under OM due to the elevated ATPase activity of myosin [18]. Beside the hemodynamic effects of OM, one has to consider the possibility that increased oxygen consumption induced by OM contributes to the mimicked ischemic preconditioning effect.

It should be noted that approximately 82% of OM is bound to plasma proteins [19], making a comparison of clinical data (angina pectoris complaints at a concentration of 3 µM and more) and in vitro results difficult.

The detected increase in the left ventricular end-diastolic pressure with higher concentrations of OM could be explained by the shortened diastolic period with the consequential loss of relaxation and filling of the heart. As LVEDP increased to values of 80 mmHg, sufficient coronary perfusion is questionable supporting our idea of an ischemic preconditioning effect. Moreover, there is a frequency- and concentration-dependent action of OM on sarcomere shortening [20]. At lower concentrations like 1 µM, the fractional shortening was increased, whereas at 10 μM this increase seemed to be reversed. The augmentation of fractional shortening at 1 µM was only seen at lower frequencies (0.5–1 Hz), whereas higher frequencies (2–3 Hz) provided a reduction in fractional shortening [21]. Whether the hemodynamic influence of OM is cardioprotective or whether a signalling pathway is activated was inconclusive from our present results. A hint for activation of a signalling pathway is the results from OM-induced postconditioning where we also detected a pronounced infarct size reduction without impaired hemodynamics. Thus, the administration of OM at the onset of reperfusion might trigger a signalling pathway leading to cardioprotection. 

A study by Szentandrassy et al. examined the electrophysiological effects of OM in canine ventricular cardiomyocytes [22]. The authors showed that OM at 10 µM led to a more positive membrane potential during the early plateau phase, and this was associated with altered electrophysiological properties of the isolated ventricular cells. Lower concentrations did not show such changes, and this is consistent with our current results where only a concentration above 10 µM had a protective effect on cardiomyocytes. Szentandrassy et al. cannot answer the question of whether these changed electrophysiological properties were due to the direct action of OM on the ion channels or due to the altered myosin–actin interaction. Future studies should investigate whether OM is protective because it modifies functional processes and also hemodynamics, or whether OM has a direct effect on corresponding protective receptors or signalling pathways. 

### Limitations

The myosin activator OM was developed for improving cardiac function in patients with heart failure. In the present study, we investigated the cardioprotective effects of OM in the healthy heart and not in the failing heart. While this is a limitation of the study, the aim was to describe the cardioprotective properties of OM per se. Furthermore, the concentrations of OM in our study resulting in infarct size reduction are supratherapeutic and in a range that provokes side effects, i.e., angina pectoris [17].

## 5. Conclusions

The results of the present study demonstrate that OM induces strong infarct size reduction by preconditioning and postconditioning in the rat heart in vitro. The exact underlying mechanisms responsible for these cardioprotective effects have to be determined in future studies.

## Figures and Tables

**Table 1:** Weights and ischemic contracture.

	*n*	Body Weight (g)	Heart Weight Dry (g)	Heart Weight Wet (g)	Time of Max. Ischemic Contracture (min)	Level of Max. Ischemic Contracture (mmHg)
Con	8	280 ± 23	0.11 ± 0.01	1.25 ± 0.12	16 ± 1	81 ± 15
OM1	8	273 ± 33	0.11 ± 0.01	1.22 ± 0.13	16 ± 2	72 ± 14
OM3	8	302 ± 31	0.12 ± 0.01	1.33 ± 0.18	21 ± 5 ^#^	60 ± 8 ^#^
OM10	8	302 ± 34	0.11 ± 0.02	1.32 ± 0.11	23 ± 3 ^#^	45 ± 9 ^#^
OM30	6	317 ± 34	0.13 ± 0.01	1.34 ± 0.13	22 ± 3 ^#^	42 ± 9 ^#^

Data are mean ± SD. Con = Control; OM = Omecamtiv mecarbil. ^#^ *p* < 0.05 vs. Con and OM1.

**Table 2:** Weights and ischemic contracture.

	*n*	Body Weight (g)	Heart Weight Dry (g)	Heart Weight Wet (g)	Time of Max. Ischemic Contracture (min)	Level of Max. Ischemic Contracture (mmHg)
Con	8	316 ± 17	0.14 ± 0.02	1.42 ± 0.12	19 ± 6	68 ± 14
OM3	8	306 ± 20	0.14 ± 0.01	1.38 ± 0.15	18 ± 5	67 ± 22
OM10	8	299 ± 24	0.13 ± 0.01	1.29 ± 0.13	18 ± 2	63 ± 21

Data are mean ± SD. Con = Control; OM = Omecamtiv mecarbil.

**Table 3:** Hemodynamic variables.

	Baseline	PC	Reperfusion	
			30	60
Heart Rate (bpm)				
Con	309 ± 40	314 ± 31	275 ± 39	272 ± 29
OM1	330 ± 39	314 ± 55	287 ± 61	261 ± 54
OM3	328 ± 29	313 ± 27	281 ± 56	284 ± 53
OM10	332 ± 45	292 ± 44	230 ± 56	287 ± 42
OM30	320 ± 55	295 ± 62	222 ± 92	275 ± 66
LVEDP (mmHg)				
Con	4 ± 1	5 ± 2	116 ± 13 *	102 ± 9 *
OM1	5 ± 1	14 ± 9	113 ± 17 *	98 ± 13 *
OM3	5 ± 1	68 ± 17 *^,#^	96 ± 30 *	83 ± 25 *
OM10	6 ± 1	79 ± 8 *^,#^	85 ± 19 *^,#^	72 ± 15 *^,#^
OM30	4 ± 1	79 ± 8 *^,#^	82 ± 25 *^,#^	76 ± 20 *^,#^
Coronary flow (mL/min)				
Con	13 ± 2	12 ± 2	7 ± 1 *	6 ± 2 *
OM1	14 ± 2	16 ± 3 ^#^	7 ± 2 *	6 ± 1 *
OM3	14 ± 3	14 ± 3 ^#^	9 ± 2 *	8 ± 2 *
OM10	16 ± 2 ^#^	11 ± 2	10 ± 2 *^,#^	9 ± 2 *^,#^
OM30	16 ± 2 ^#^	14 ± 1	10 ± 3 *^,#^	10 ± 1 *^,#^

Con = Control; OM = Omecamtiv mecarbil. * *p* < 0.05 vs. baseline; ^#^ *p* < 0.05 vs. Con.

**Table 4:** Hemodynamic variables.

	Baseline	Reperfusion		
		30	45	60
Heart Rate (bpm)				
Con	323 ± 32	220 ± 78 *	271 ± 33	278 ± 51
OM3	320 ± 32	231 ± 51	257 ± 37	261 ± 56
OM10	312 ± 23	205 ± 55 *	211 ± 58	220 ± 72
LVEDP (mmHg)				
Con	4 ± 1	90 ± 23 *	82 ± 19 *	81 ± 19 *
OM3	5 ± 2	81 ± 27 *	78 ± 23 *	75 ± 22 *
OM10	5 ± 1	92 ± 18 *	85 ± 17 *	83 ± 14 *
Coronary flow (mL/min)				
Con	16 ± 2	9 ± 2 *	9 ± 2 *	8 ± 2 *
OM3	16 ± 2	11 ± 4 *	10 ± 4 *	9 ± 4 *
OM10	17 ± 2	11 ± 2 *	9 ± 2 *	9 ± 2 *

Con = Control; OM = Omecamtiv mecarbil. * *p* < 0.05 vs. baseline.
